# *GDF11* upregulation independently predicts shorter overall-survival of uveal melanoma

**DOI:** 10.1371/journal.pone.0214073

**Published:** 2019-03-18

**Authors:** Xun Liu, Qinghai Zhang, Chuanfeng Fan, Jie Tian, Xinchang Liu, Guofeng Li

**Affiliations:** 1 Department of Ophthalmology, Weifang People's Hospital, Weifang, China; 2 Department of ICU, Weifang People's Hospital, Weifang, China; 3 Department of Ophthalmology, Taian Aier Eye Hospital, Taian, China; University of Nebraska Medical Center, UNITED STATES

## Abstract

Growth differentiation factor 11 (GDF11), is a member of the transforming growth factor-beta (TGF-β) superfamily and bone morphogenetic protein (BMP) subfamily. In this study, we aimed to assess the expression profile of *GDF11*, its prognostic value in terms of OS, as well as the potential mechanisms leading to its dysregulation in uveal melanoma. A retrospective study was conducted using our primary data and genetic, clinicopathological and overall survival (OS) data from the Cancer Genome Atlas-Uveal Melanoma (TCGA-UVM). Results showed that *GDF11* expression was significantly higher in tumor tissues compared with that in adjacent normal tissues. High *GDF11* expression was associated with uveal melanoma in advanced stages (IV), epithelioid cell dominant subtype, as well as extrascleral extension. Univariate analysis showed that older age, epithelioid cell dominant, with extrascleral extension and increased *GDF11* expression were associated with unfavorable OS. Multivariate analysis confirmed that *GDF11* expression was an independent prognostic indicator of unfavorable OS (HR: 1.704, 95%CI: 1.143–2.540, *p* = 0.009), after adjustment of age, histological subtypes and extrascleral extension. Among the 80 cases of uveal melanoma, only 3 cases had low-level copy gain (+1) and 2 cases had heterozygous loss (-1). No somatic mutations, including SNPs and small INDELs were observed in *GDF11* DNA. The methylation of these four CpG sites had weakly (cg22950598 and cg23689080), moderately (cg09890930), or strongly (cg05511733) negative correlation with *GDF11* expression. In addition, the patients with high methylation of these four sites had significantly better OS compared to the group with low methylation. Based on these findings, we infer that methylation modulated *GDF11* expression might be a valuable prognostic biomarker regarding OS in uveal melanoma.

## Introduction

Uveal melanoma arises from the melanocytes residing within the uvea and is the most common primary intraocular cancer in adults [1]. The risk of metastasis and death varied significantly among patients with different stages of tumor. For stage III tumors, the metastasis and death rates at five years were 44% and 27%. In comparison, all stage IV tumors had metastasis and death by one year [2]. Although long-term survival is uncommon in patients with metastatic tumors, certain subsets of patients had long-term survival [3, 4]. However, the characteristics associated with long-term survival have not been fully understood.

Growth differentiation factor 11 (GDF11), is a member of the transforming growth factor-beta (TGF-β) superfamily and bone morphogenetic protein (BMP) subfamily [5]. GDF11 transmits signals through type I and II serine/threonine kinase receptors, which is similar to other TGF-β superfamily members [6]. Therefore, GDF11 can activate both Smad and non-Smad signals via binding to its receptors and subsequently regulate the expression of the downstream genes [6]. In the past decades, a series of studies showed that GDF11 is involved in embryonic development such as spinal cord anterior/posterior patterning and the development of urogenital system [6–8]. In addition, it plays an important role in the pathologic development of some diseases, such as cardiovascular disease, diabetes mellitus, and cancer.

In cancer biology, the role of GDF11 is conflicting. In patients with colorectal cancer, GDF11 was upregulated in tumor tissues compared with adjacent normal tissues [9]. In addition, high *GDF11* expression is associated with a higher risk of lymph node metastasis and poorer overall survival [9]. In oral squamous cell carcinoma (OSCC), *GDF11* overexpression is associated with induced epithelial to mesenchymal transition (EMT), cell migration as well as metastasis [10]. In comparison, GDF11 acts as a tumor suppressor in triple-negative breast cancer (TNBC) via promoting an epithelial and anti-invasive phenotype [11] and also suppresses the proliferation, migration and invasion of pancreatic cancer cells [12].

In this study, using primary tissue data and secondary data from the Cancer Genome Atlas-Uveal Melanoma (TCGA-UVM), we assessed the expression profile of *GDF11*, its prognostic value in terms of OS, as well as the potential mechanisms leading to its dysregulation.

## Patients and methods

The study was approved by the ethics committee of Weifang People's Hospital, China. Written informed consent was obtained from all patients before this study.

### Specimens

Tumor samples and their adjacent morphologically normal tissues (> 0.3 cm from the tumor) were obtained from 19 uveal melanoma patients who received primary enucleation and without prior radiation or chemotherapy, at the Department of Ophthalmology, Weifang People's Hospital, China. The tissues were snap-frozen in nitrogen and stored at −80°C before RNA extraction. All patients were given diagnoses according to current ophthalmologic criteria. Tumor diameter varied from 8 mm to 15 mm (mean±SD: 10.8±1.7 mm). Histopathological analysis showed that 6 tumors were epitheloid, 10 were spindle cell, and 3 were mixed histology.

### Quantitative real-time PCR (qPCR) assay

Total RNA was extracted from tissue Samples using TRIzol reagent (Invitrogen, Carlsbad, CA, USA) according to the manufacturer’s instruction. RNA was reversely transcribed into cDNA using SuperScript III First-Strand Synthesis SuperMix (Invitrogen). Then, qPCR was performed using SYBR Premix Ex Taq II (TaKaRa), with the Applied Biosystems 7500 Real-Time PCR System (Applied Biosystems, Foster City, CA, USA). The specific primers used to detect *GDF11* were as follows: Forward, 5'-GCAAGTGCTACACAGCTGGTTC-3' and reverse, 5'‑CTCTAGGACTCGAAGCTCCATG‑3'. The specific primers used for β-actin were as follows: Forward, 5'-GAAGAGCTACGAGCTGCCTGA-3' and reverse, 5'-CAGACAGCACTGTGTTGGCG-3'. β-actin was used as an internal reference. Experiments were performed in triplicate. Gene expression was normalized to β-actin using the 2^-ΔΔCT^ method.

### Infinium HumanMethylation450 BeadChip assay

Genomic DNA (gDNA) methylation status of the primary tissue samples was examined using the Infinium HumanMethylation450 BeadChip kit (450K) (Illumina, San Diego, CA, USA) according to manufacturer’s instructions. Briefly, gDNA was extracted from the tumor and adjacent normal tissues and was treated using the EZ DNA Methylation kit (Zymo Research, Orange, CA, USA) for bisulfite modification. The samples after purification were subjected to hybridization on Infinium HumanMethylation450 (450K) BeadChips, following the protocol recommended by the manufacturer. The signal intensities and the methylation level of each CpG site were determined using the GenomeStudio software. Beta values were calculated following the formula: β = M/(U+M+100), in which M refers to the fluorescence level of the methylation probe and U means the methylation level of the unmethylated probe.

### Data mining in the cancer Genome Atlas-Uveal melanomas (UVM)

The level 3 data in TCGA-UVM were downloaded by using the UCSC Xena browser (https://xenabrowser.net). In this dataset, 80 patients with primary uveal melanomas, who had no history of neoadjuvant treatment were included. The basic information of the patients can be obtained from: https://portal.gdc.cancer.gov/projects/TCGA-UVM.

Their clinicopathological and survival data including age at diagnosis, gender, histological type, pathological stage, pathological nodal status, pathological metastasis status, tumor diameter (mm), tumor thickness (mm), extrascleral extension and OS status and OS in days were downloaded for survival analysis. Among the 80 patients included, 23 cases were deceased by the end of the project, among which 19 cases were due to metastatic uveal melanoma.

To explore the mechanisms of *GDF11* dysregulation in uveal melanoma, the genetic data including DNA copy number alterations (CNAs), somatic mutation, as well as *GDF11* DNA methylation were also downloaded via the UCSC Xena browser (https://xenabrowser.net/). CNAs were calculated by gene-level thresholded Genomic Identification of Significant Targets in Cancer 2.0 (GISTIC2), which defines CNAs as homozygous deletion (-2), heterozygous loss (-1), copy-neutral (0), low-level copy gain (+1), high-level amplification (+2) were downloaded from the Xena browser. Somatic mutation data included single nucleotide polymorphisms (SNPs) and small insertions and deletions (INDELs). *GDF11* DNA methylation was measured by Illumina Infinium Human Methylation 450K BeadChip.

### Statistical analysis

*GDF11* expression in different groups was compared using one-way ANOVA followed by Tukey post-hoc test or using Welch’s *t*-test. Kaplan-Meier curves of overall survival (OS) were generated by GraphPad Prism v6.0 (GraphPad Software Inc., La Jolla, CA, USA). Two types of grouping were performed to separate the patients: 1, based on median *GDF11* expression irrespective of methylation status, 2, strictly based on *GDF11* methylation status irrespective of its expression status. Receiver operating characteristic (ROC) curve of *GDF11* expression for death detection was plotted and area under the curve (AUC) was calculated. According to the AUC values, the accuracy of a prognostic test can be roughly classified into five categories: 0.90–1 = excellent, 0.80–0.90 = good; 0.70–0.80 = fair; 0.60–0.70 = poor and 0.50–0.60 = fail [13]. The association between *GDF11* expression and the clinicopathological parameters was examined by using χ^2^ test by two-sided Fisher’s exact test. The difference between the survival curves was assessed using the Log-rank test. Univariate and multivariate Cox regression models were applied to evaluate the independent prognostic value of *GDF11*, as a continuous variable. Linear regression analysis was conducted to evaluate the correlation between *GDF11* expression and the methylation value of each CpG sites. *p* < 0.05 was considered statistically significant.

## Results

### *GDF11* expression profile in uveal melanoma

**Fig 1:**
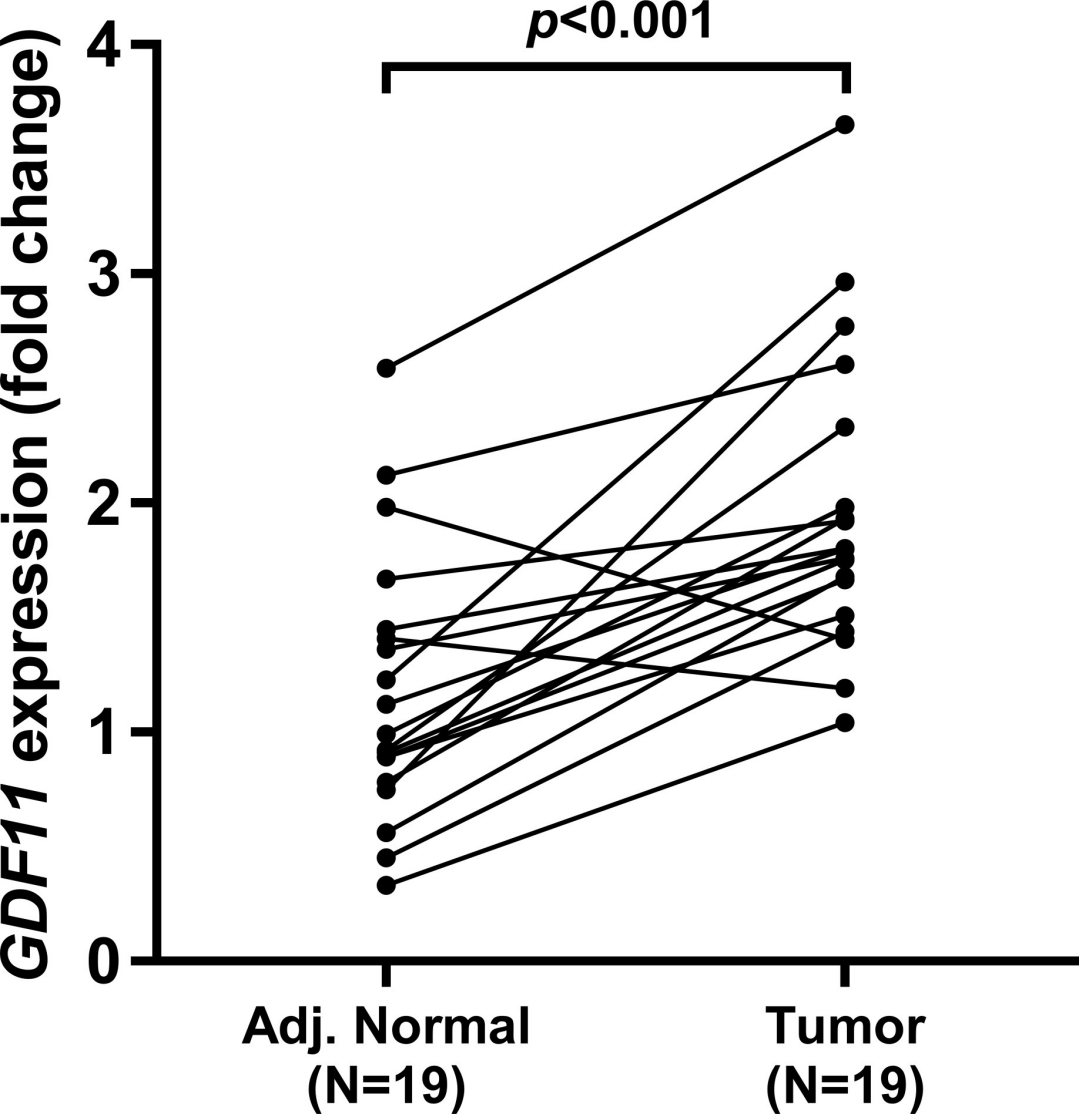
*GDF11* expression in uveal melanoma and adjacent normal tissues. qPCR analysis of *GDF11* expression in 19 cases of uveal melanoma and adjacent normal tissues. 2^-ΔΔCT^ method was used to assess relative *GDF11* expression.

**Fig 2:**
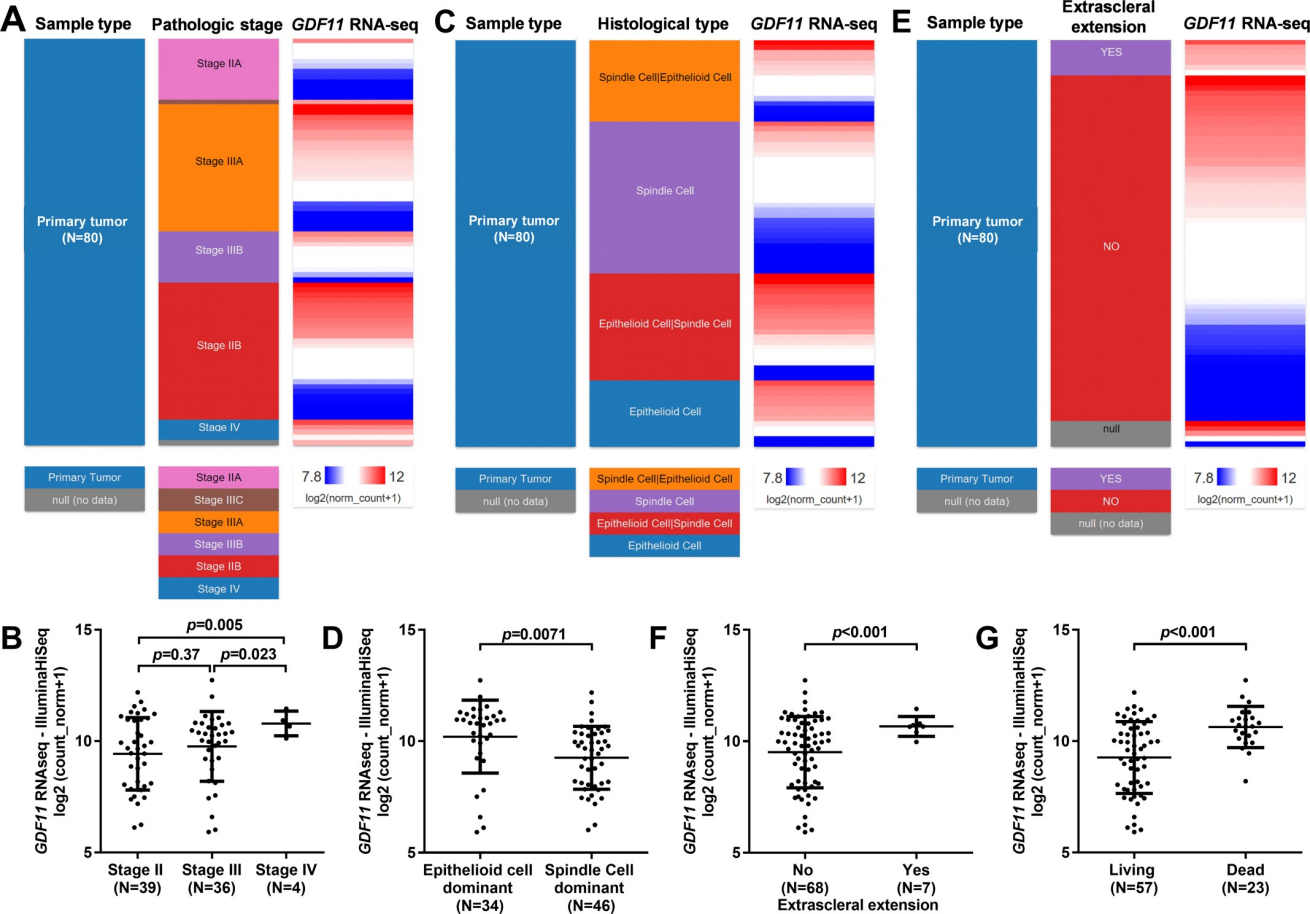
*GDF11* expression profile in uveal melanoma. **A-F.** Heatmaps (A, C and E) and plot charts (B, D and F) showing the comparison of *GDF11* expression in different pathological stages (A-B), between epithelioid cell dominant and spindle cell dominant subtypes (C-D) and between cases with or without extrascleral extension (E-F). **G.** Comparison of GDF11 expression between deceased and living UVM patients. Original data were obtained from TCGA-UVM.

By performing qPCR assay, we examined *GDF11* expression in 19 cases of uveal melanoma tumor tissues and adjacent normal tissues. Results showed that *GDF11* was generally upregulated in the tumor tissues compared with adjacent normal tissues (Fig 1). To further explore the association between *GDF11* expression and the phenotypes of uveal melanoma, we retrieved RNA-seq and clinicopathological data in TCGA-UVM. By grouping the tumor cases according to pathological stages, we found that stage IV tumors had the highest *GDF11* expression (Fig 2A and 2B). In comparison, the difference between stage II and stage III tumors was not significant (Fig 2A and 2B). By comparing *GDF11* expression between epithelioid cell dominant and spindle cell dominant subtypes, we found that the more malignant epithelioid cell dominant subtype had significantly higher *GDF11* expression (Fig 2C and 2D). In addition, we also found that the samples with extrascleral extension had significantly upregulated *GDF11* expression compared to the negative cases (Fig 2E and 2F). Notably, *GDF11* expression was substantially higher in the deceased cases compared with that in the living cases (Fig 2G).

### Increased *GDF11* expression independently predicts unfavorable OS in uveal melanoma

**Fig 3:**
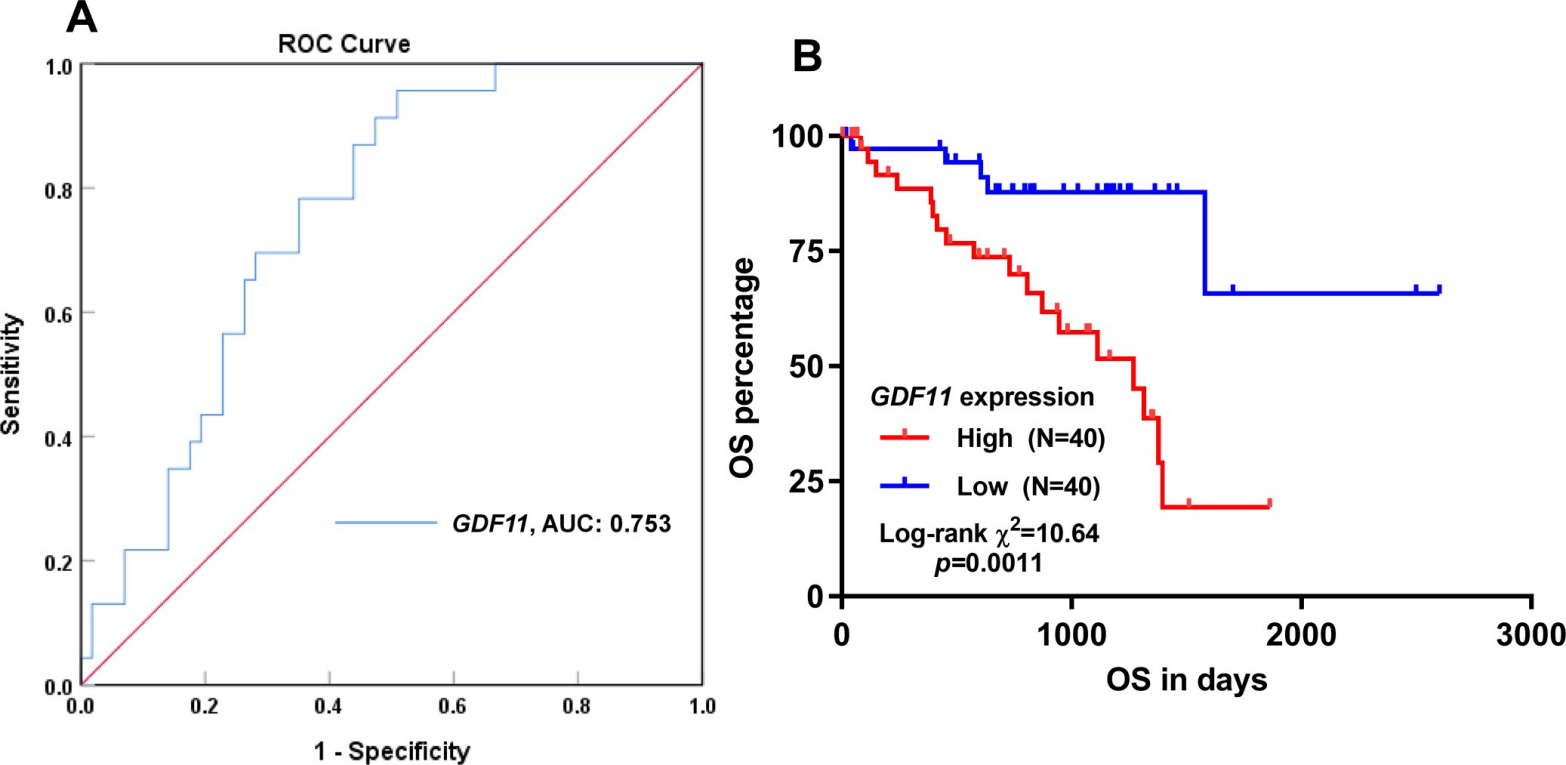
Kaplan-Meier curves of OS in patients with uveal melanoma. **A.** ROC curves and AUC statistics to evaluate the prognostic value of *GDF11* expression regarding to death in UVM patients. **B.** Kaplan-Meier curves of OS in patients with uveal melanoma. Patients were grouped according to median *GDF11* expression. Original data were obtained from TCGA-UVM.

By performing ROC analysis regarding OS, *GDF11* expression had an AUC value of 0.753 (Fig 3A), suggesting that high *GDF11* expression might be a fair marker of unfavorable OS. Then, Kaplan-Meier curves of OS were generated by setting median *GDF11* expression as the cutoff. Results that the patients with high *GDF11* expression had significantly shorter OS, compared to the patients with low *GDF11* expression (*p* = 0.001, Fig 3B). By comparing the clinicopathological parameters between the high and low *GDF11* expression groups, we found that the high expression group was significantly older (mean ± SD, 64.7±12.45 *vs*. 58.60 ± 14.83, *p* = 0.05), had a higher proportion of epithelioid cell dominant subtype (25/40 *vs*. 9/40, *p* < 0.001), more patients in advanced stages, thicker tumors (> 10 mm *vs*. ≤ 10 mm, 27/40 *vs*. 16/40, *p* = 0.024) and a higher death rate (18/40 *vs*. 5/40, *p* = 0.003) (Table 1). By performing univariate analysis, we found that older age, epithelioid cell dominant, with extrascleral extension and increased *GDF11* expression were associated with unfavorable OS (Table 2). In multivariate analysis, *GDF11* expression was an independent prognostic indicator of unfavorable OS (HR: 1.704, 95%CI: 1.143–2.540, *p* = 0.009) (Table 2).

**Table 1:** The association between *GDF11* expression and the clinicopathological parameters in patients with primary uveal melanoma in TCGA.

Parameters		*GDF11* expression		*p* value
		High (N = 40)	Low (N = 40)	
Age (Mean ± SD)		64.7 ± 12.45	58.60 ± 14.83	0.05
Gender	Female	16	19	0.65
	Male	24	21	
Histological type	Epithelioid cell dominant	25	9	<0.001
	Spindle Cell dominant	15	31	
Pathological Stage	II	14	25	0.012
	III	21	15	
	IV	4	0	
	Null	1	0	
Pathological N	N0	27	25	N.A.
	NX	12	15	
Pathological M	M0	26	25	0.12
	M1/M1b	4	0	
	MX + null	10	15	
Tumor diameter (mm)	> 16	24	21	0.50
	≤ 16	15	19	
	Null	1	0	
Tumor thickness (mm)	> 10	27	16	0.024
	≤ 10	13	24	
Extrascleral extension	No	31	37	0.056
	Yes	6	1	
	Null	3	2	
Living Status	Living	22	35	0.003
	Dead	18	5	

Extrascleral extension: extension occurring outside the sclera of the orbit. NX: Nearby (regional) lymph nodes cannot be assessed; null: data were not available; N/A: not applicable.

**Table 2:** Univariate and multivariate analyses of OS in patients with primary uveal melanoma.

Parameters	Univariate analysis				Multivariate analysis			
	*p*	HR	95%CI (lower/upper)		*p*	HR	95%CI (lower/upper)	
**Age** (Continuous)	**0.019**	1.046	1.008	1.085	0.069	1.039	0.997	1.082
**Female** *vs*. **Male**	0.325	0.649	0.274	1.536				
**Histological type** Epithelioid cell dominant *vs*. Spindle Cell dominant	**0.001**	4.551	1.814	11.418	0.055	2.628	0.978	7.064
**Pathological stage** III/IV *vs*. II	0.358	1.504	0.630	3.589				
**Tumor diameter (mm)** >16 *vs*. ≤16	0.192	1.831	0.738	4.541				
**Tumor thickness (mm)** >10 *vs*. ≤10	0.106	2.106	0.854	5.191				
**Extrascleral extension** No *vs*. Yes	**0.008**	0.219	0.071	0.675	0.135	0.388	0.112	1.344
***GDF11* expression** (Continuous)	**<0.001**	1.915	1.348	2.721	**0.009**	1.704	1.143	2.540

### Genetic and epigenetic related mechanisms underlying the dysregulation of *GDF11* in uveal melanoma

**Fig 4:**
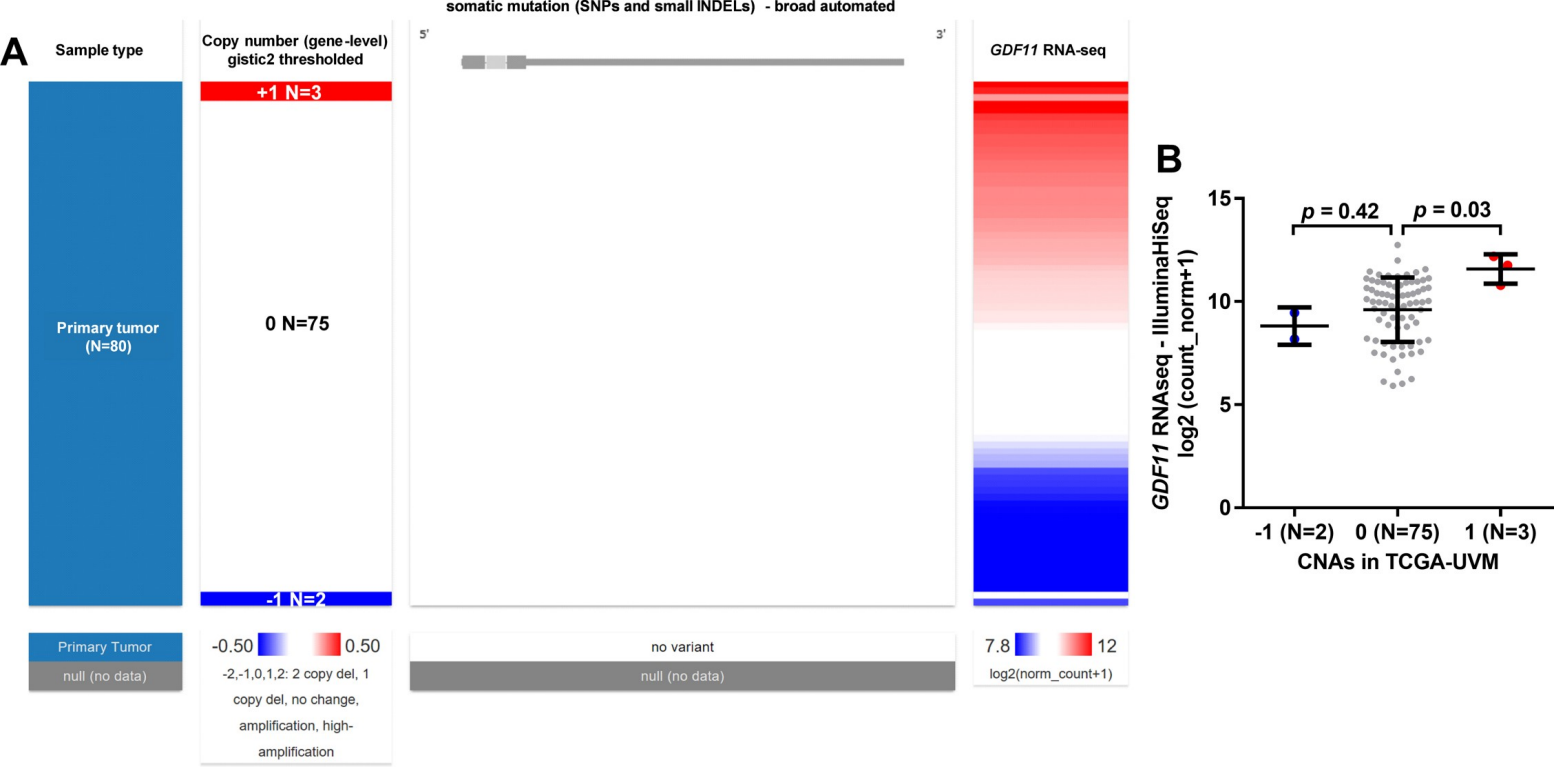
Genetic alterations of *GDF11* DNA in uveal melanoma. **A.** Heatmap showing the correlation between *GDF11* expression and its DNA CNAs/somatic mutations. **B.** Plots chart showing the expression of *GDF11* in different CNA groups. Original data were obtained from TCGA-UVM.

Since we identified that *GDF11* expression might be a valuable prognostic indicator in uveal melanoma, we then tried to assess the potential mechanisms of its dysregulation. By examining *GDF11* DNA CNAs in uveal melanoma, we found that DNA amplification and deletion were not frequent. Among the 80 cases of uveal melanoma, only 3 cases had low-level copy gain (+1) and 2 cases had heterozygous loss (-1) (Fig 4A). However, although the low-level copy gain cases had significantly elevated *GDF11* expression compared to the copy neutral (0) cases, the *GDF11* heterozygous loss did not necessarily result in *GDF11* downregulation (Fig 4B). In addition, no somatic mutations, including SNPs and small INDELs were observed in *GDF11* DNA (Fig 4A), suggesting that *GDF11* dysregulation was less likely to be influenced by genetic alteration.

**Fig 5:**
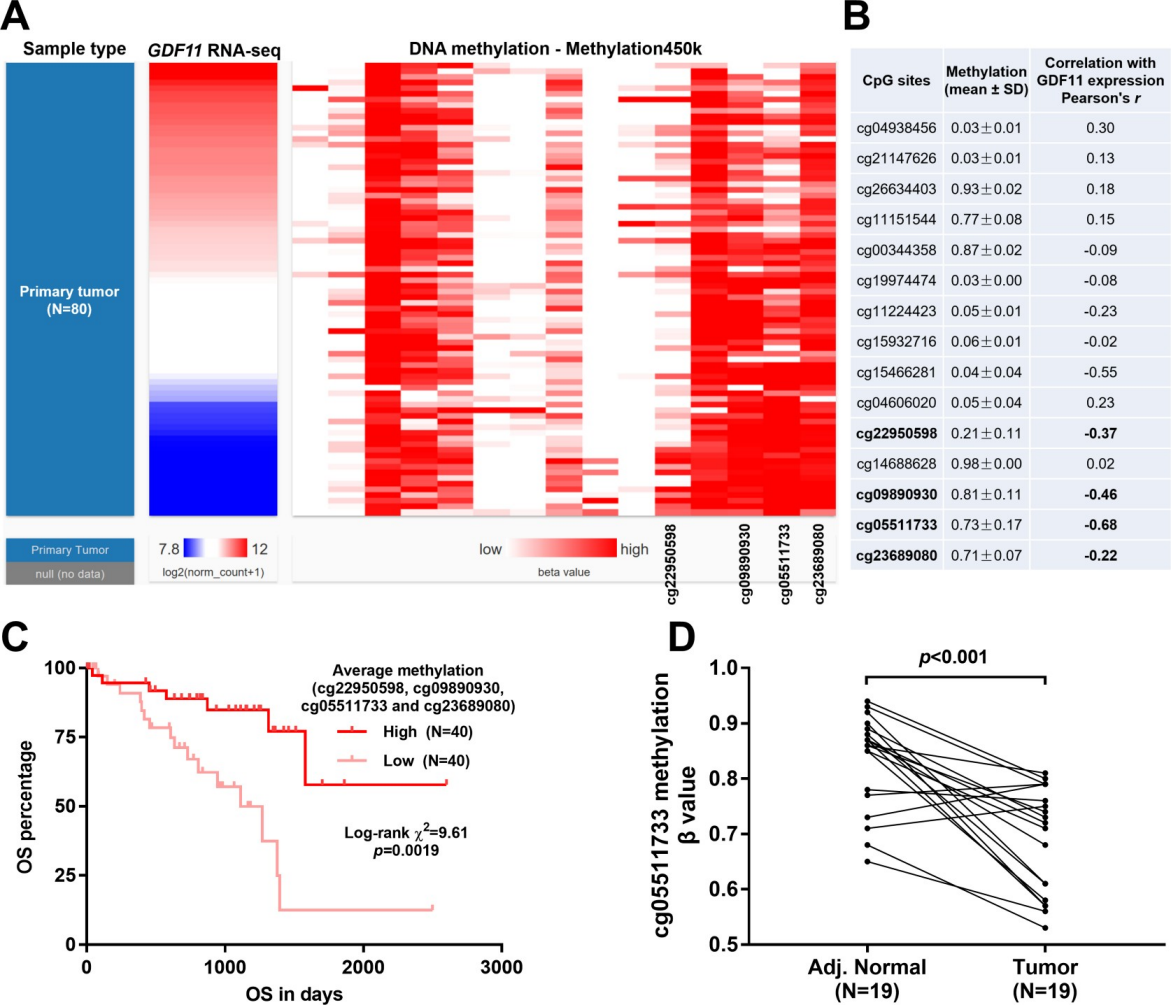
Methylation modulated *GDF11* expression was a valuable prognostic biomarker in uveal melanoma. **A.** Heatmap showing the correlation between *GDF11* expression and the methylation status of 15 CpG sites in uveal melanoma. **B.** Summary of the correlations between *GDF11* expression and the methylation status of 15 CpG sites. **C.** Kaplan-Meier curves of OS in patients with uveal melanoma. Patients were grouped according to median methylation of cg22950598, cg09890930, cg05511733 and cg23689080. Original data was obtained from TCGA-UVM. **D.** Comparison of the β value of cg05511733 methylation level in 19 primary tumor and adjacent normal tissues.

Then, we further explored the association between *GDF11* expression and its DNA methylation, an epigenetic mechanism influencing gene expression. The methylation status of 15 CpG sites in *GDF11* DNA was measured in Illumina Infinium Human Methylation 450K BeadChip. In the heatmap, we found that the methylation of some CpG sites (cg22950598, cg09890930, cg05511733 and cg23689080) were negatively correlated with *GDF11* expression (Fig 5A). By performing linear regression analysis, we confirmed that the methylation of these four CpG sites had weakly (cg22950598 and cg23689080), moderately (cg09890930), or strongly (cg05511733) negative correlation with *GDF11* expression (Fig 5B). Although another CpG site cg15466281 also showed a moderately negative correlation with *GDF11* expression (Pearson’s r = -0.55), the average methylation of this site was low in uveal melanoma (mean ± SD: 0.04 ± 0.04) (Fig 5B). Therefore, the influence of this site on *GDF11* expression was limited.

Then, we assessed the association between the average methylation of cg22950598, cg09890930, cg05511733 and cg23689080 and OS of uveal melanoma. Kaplan-Meier showed that the group with high methylation had significantly better OS compared to the group with low methylation (Fig 5C). This finding further confirmed that methylation modulated *GDF11* expression was a valuable prognostic biomarker in uveal melanoma. Since the status of cg05511733 was strongly and negatively correlated with *GDF11* expression, we compared the methylation level of this CpG site between the 19 primary tumor and adjacent normal tissues. Results showed that the adjacent normal group had a significantly higher level of methylation than the tumor group (p<0.001, Fig 5D).

## Discussion

In this study, by using data from TCGA-UVM, we demonstrated that high *GDF11* expression was associated with uveal melanoma in advanced stages (IV), epithelioid cell dominant subtype, as well as extrascleral extension. More importantly, we confirmed that *GDF11* expression was an independent prognostic indicator of unfavorable OS (HR: 1.704, 95%CI: 1.143–2.540, *p* = 0.009), after adjustment of age, histological subtypes and extrascleral extension. These findings suggest that *GDF11* might serve as a valuable prognostic biomarker in uveal melanoma.

GDF11 firstly binds to Activin receptor II (ActRIIA and ActRIIB), and then recruits Activin receptor I (ActRI) including activin receptor-like kinase 1 (ALK1), ALK4, ALK5 and ALK7 [14, 15]. After that, their complex activates canonical Smad signaling via including Smad2/3 and Smad1/5/8 [6]. Besides, the complex can also activate non-Smad signals such as Rho-like GTPase, MAP kinases (including p38, ERK and JNK), and phosphatidylinositol-3-kinase/AKT [16]. Several recent studies found that GDF11 acts an important stimulator of angiogenesis. It stimulates angiogenesis via in focal cerebral ischemia/reperfusion rats via ALK5 [17], and also stimulates pulmonary artery endothelial cell (PAEC) proliferation, migration, tube formation, via activating ALK1/p-Smad1/5/8 and ALK5/p-Smad2/3 signals [18]. Angiogenesis plays a critical role in the progression and metastasis of uveal melanoma [19, 20]. Inhibition of angiogenesis via the neddylation pathway inhibited hepatic metastasis in uveal melanoma, using NOD-SCID mouse xenograft model [21]. These mechanisms help to explain the association between GDF11 upregulation and the poor OS of uveal melanoma. Although some studies reported that GDF11 might be a tumor suppressor in some other cancers, such as TNBC [11], the contradictory results might be a result of the dual role of TGF-β in the different stages of cancer. In normal cells and early carcinomas, TGF-β signaling pathways mainly exerts tumor suppressive effect. However, the protective effects of TGF-β signaling are usually lost, which in turn switches to promote tumor progression, invasion and metastasis [22].

The mechanisms underlying *GDF11* dysregulation were quite complex in different diseases and might be tissue specific. In BALB/c-3T3 cells, *GDF11* expression is activated by the histone deacetylase (HDAC) inhibitor trichostatin A, while is repressed by HDAC3 [23]. In PAECs, the transcription factor zinc finger protein 740 directly binds to the *GDF11* promoter and enhances its transcription [18]. In this study, we explored the potential genetic and epigenetic (typically methylation) alterations in *GDF11* DNA in uveal melanoma. Results showed that DNA CNAs were not frequent in uveal melanoma. In addition, no somatic mutations, including SNPs and small INDELs were observed in *GDF11* DNA. However, we found that that the methylation of these four CpG sites had weakly (cg22950598 and cg23689080), moderately (cg09890930), or strongly (cg05511733) negative correlation with *GDF11* expression. Also, we demonstrated that the patients with high methylation of these four sites had significantly better OS compared to the group with low methylation. In addition, using data from primary samples, we confirmed that the adjacent normal group had a significantly higher level of cg05511733 methylation than the tumor group. These findings indicated that methylation is an important mechanism of *GDF11* dysregulation in uveal melanoma.

This study also has some limitations. Firstly, we only explored the association between *GDF11* expression in tumor tissues and the OS of uveal melanoma patients. In the future, it is quite necessary to explore whether it has prognostic value as a circulating protein found in serum. In addition, since only OS data was recorded in TCGA-UVM, we had not evaluated the prognostic value regarding disease-free survival (DFS). This also needs to be assessed in a large patient cohort in the following studies.

## Conclusion

Methylation modulated *GDF11* expression might be a valuable prognostic biomarker in terms of OS in uveal melanoma.
